# Effects of structured written feedback by cards on medical students’ performance at Mini Clinical Evaluation Exercise (Mini-CEX) in an outpatient clinic

**Published:** 2016-07

**Authors:** FARIBA HAGHANI, MOHAMMAD HATEF KHORAMI, MOHAMMAD FAKHARI

**Affiliations:** 1 Department of Medical Education, Medical Education Research Center, Isfahan University of Medical Sciences, Isfahan, Iran;; 2 Department of Urology, Isfahan University of Medical Sciences, Isfahan, Iran;; 3 Medical Education Research Center, Isfahan University of Medical Sciences, Isfahan, Iran

**Keywords:** Feedback, Medical education, Ambulatory care, Outpatient clinics

## Abstract

**Introduction:**

Feedback cards are recommended as a feasible tool for structured written feedback delivery in clinical education while effectiveness of this tool on the medical students’ performance is still questionable.  The purpose of this study was to compare the effects of structured written feedback by cards as well as verbal feedback versus verbal feedback alone on the clinical performance of medical students at the Mini Clinical Evaluation Exercise (Mini-CEX) test in an outpatient clinic.

**Methods:**

This is a quasi-experimental study with pre- and post-test comprising four groups in two terms of medical students’ externship. The students’ performance was assessed through the Mini-Clinical Evaluation Exercise (Mini-CEX) as a clinical performance evaluation tool. Structured written feedbacks were given to two experimental groups by designed feedback cards as well as verbal feedback, while in the two control groups feedback was delivered verbally as a routine approach in clinical education. **

**Results:**

By consecutive sampling method, 62 externship students were enrolled in this study and seven students were excluded from the final analysis due to their absence for three days. According to the ANOVA analysis and Post Hoc Tukey test,  no statistically significant difference was observed among the four groups at the pre-test, whereas a statistically significant difference was observed between the experimental and control groups at the post-test  (*F *= 4.023, *p =0.012*). The effect size of the structured written feedbacks on clinical performance was 0.19.

**Conclusion:**

Structured written feedback by cards could improve the performance of medical students in a statistical sense. Further studies must be conducted in other clinical courses with longer durations.

## Introduction

Clinical education is a major part of medical education which allows the students to apply their theoretical knowledge in practice within a clinical setting. Providing effective feedback is a well-known educational strategy to enhance the effectiveness of the clinical teaching-learning process (1, 2). Clinical instructors deliver verbal feedback to the students as a routine approach. To enhance the effeciency, some experts believe verbal feedback is a weak method of feedback delivery due to the fact that it is easily forgotten by students and instructors and decreases the probability of re- observation and reflection (3-5). Written feedback overcomes these limitations. Furthermore, it has some more advantages compared to verbal feedback.  With written feedback, students are less likely to misinterpret or forget the feedback content; it provides a common perception on feedback content between instructors and students and enhances the students’ reflection on the delivered feedback content (6-9).

During the recent years, researchers have focused on developing practical tools for providing written and documented feedbacks to the learners without interrupting the educational process in clinical settings. Among these tools, feedback cards are the most favorable means for delivering the structured written feedback (5,7). Feedback cards have numerous advantages due to their small size and easy handling. Furthermore, it could be applied in most educational settings without any interruption on the educational process (10). Although some studies have reported the learners’ satisfaction with this feedback provisional method, the effectiveness of these cards on the clinical performance of the students is still questionable (11-14). Previous studies highlight the importance of experimental studies on the effectiveness of feedback tools on clinical performance (15, 16).

Clinical performance is a complex subject to measure.  Mini Clinical Evaluation Exercise (Mini-CEX) is a highly recommended tool for evaluating the performance of undergraduate medical students in most clinical settings (17-18). It is being adopted as a feasible, valid and reliable tool to evaluate six main clinical skills (19). This study investigated the effect of structured written feedback by cards on medical students’ performance at the Mini-CEX test in an outpatient clinic.

## Methods

This is a quasi-experimental study with pre- and post-test comprising four groups in two terms of medical students’ externship. This study was conducted in an educational hospital of Isfahan University of Medical Sciences in Iran.  

The medical students in two educational terms (the first was related to the February to April 2014 term and the second to the May to July 2014 term) participated in this study. Consecutive sampling method was adopted here where all externship students (30 students in the first term and 32 in the second term) were enrolled in this study. All the students signed the written consent to participate in the study. Participants were allocated into four groups (two from each term) based on predetermined students’ list of names in the course schedule. The researchers entered the first group in each term as the control group in the study to decrease the probability of the diffusion of the intervention of the study process. After allocation, the first group (*n *= 15) and the third group (*n *= 17) were used as the control groups while the second group (*n *= 14) and the fourth group (*n *= 15) were used as the experimental groups. During this study, 3 students in the first term and 5 in the second term were excluded due to their absence for three days. Data of 54 students were analyzed finally.  Two clinical instructors examined the performance of students by Mini-CEX test as pre- and post-test. The intra-rater reliability of Mini-CEX test was controlled by assigning the same instructors for pre- and post-test in each group.

The instructors delivered verbal feedbacks to the two control groups during the outpatient clinical teaching sessions. The two experimental groups received both verbal feedbacks and written feedback simultaneously. The feedback card was designed to deliver the structured written feedback in the two experimental groups based on the recommended format in previous studies (10-12,20, 21). The feedback card was adjusted for outpatient clinic performance by four medical education experts and clinical instructors. These cards were 10×13 cm in size.  In order to deliver the structured written feedback, each card had a table with eight performance items and  scoring boxes for rating the performance of students as good, satisfactory, or dissatisfactory by the instructors, as presented in previous studies. The back of the card had sufficient space for additional feedback notes.

The Mini-Clinical Evaluation Exercise (Mini-CEX) is an adopted tool for measurements of clinical performance in a wide variety of clinical settings. This test was utilized to evaluate the ability of learners in six main skills related to assessment and management of patients in real conditions (17-19). The Mini-CEX offered on single sheet is more feasible to carry out, easy to handle and time saving in a busy clinical setting.  The Mini-CEX test sheet consists of three parts: the first part contained three questions on patient characteristics (age, gender, and previous visits at the clinic); in the second part, a table was used to rate the complexity of cases (from low to high); also, in the third part, the performance of students in six domains was scored. They are medical interview, physical examination, professionalism, clinical judgment, data organization/sufficiency, and counseling skills. These six skills are scored on a scale of 0-9 (0: absence of the desired behavior, 1-3: below the expectations for the level of training, 4-6: meets the expectations and 7- 9:  above the expectations for the level of training). The total scores of the checklist, ranging from 0 to 54, are regarded as the students’ performance score (22). The content and face validity of this test have been confirmed in previous studies (19, 23, 24). Consistency of the Mini-CEX scores increased in multiple measurements (25). The Mini-CEX test had an inter-class correlation (ICC) lower than 50% and G coefficient in a single assessment of a student (18, 25).

During the pre- and post-tests, each student chooses a volunteer patient in outpatient clinic and begins to take his/her medical history, perform physical examination, and determine some possible differential diagnoses. In the next step, the students present their selected patient to the instructor at the outpatient clinic in the presence of other students. In the final steps, the instructor completes the patient’s assessment, and determines treatment planning and discharge for the patient. The instructor completes the Mini-CEX checklist immediately to prevent the interference between patient visit and student performance evaluation. Furthermore, the Mini-CEX test results were not used as the students’ final course evaluation and the confidentiality of the participants was respected by collection and saving of the test sheets.

A self-report questionnaire was completed at the end of the course to assess the students’ opinion on the quantity and quality of the delivered feedbacks. This questionnaire consisted of seven items, scored on a five-point scale of 1 to 5; from strongly disagree as 1  to strongly agree as 5. In the experimental group, the self-report questionnaire contained five additional items regarding their opinions on the utilization of feedback cards. The results of these five items were analyzed separately. The face validity of this checklist was approved by four instructors from the Department of Medical Education and the reliability of the questionnaire was acceptable based on Cronbach’s alpha test (r = 0.74).

 The Pearson correlation, the Cross Tab Chi-Square test and the One Way Analysis of Variance (ANOVA) with Post Hoc Tukey test were used to compare the data among the four groups. The significance level of difference between the groups was considered p<0.05 in the data analysis. All analyses were performed in SPSS, version 14. 

**Ethical consideration**

This study was performed in accordance with the Declaration of Helsinki and subsequent revisions and approved by the Medical Ethics Committee of the Isfahan University of Medical Sciences.  

## Results

The data of 54 students were analyzed. According to ANOVA contrast analysis among the four groups, there were no significant differences in student age (*F*=1.46, *p=0.23*); also, Chi Square test results revealed that the proportion of the students’ gender was similar (**χ**2=1.51, *p=0.68*). There was no significant difference in demographic variables of the patients among the four groups. ANOVA contrast analysis revealed similarity in patients’ age among the four groups (*F*=0.27, *p=0.84*).  Chi Square results revealed that the proportions of the patients’ gender (**χ**2=1.35, *p=0.71*), previous visits at the clinic (**χ**2= 5.33, *p=0.14*) and complexity of the case (**χ**2=9.76, *p=0.13*) were similar among the four groups. Also at the post-test, there were no significant differences in patients’ age (*F*=1.27, *p=0.29*), gender (**χ**2=6.33, *p=0.09*), previous visits at the clinic (**χ**2=4.66, *p=0.19*) and complexity of the case (**χ**2=0.35, *p=0.94*).

Analysis of Mini-CEX scores at post-test revealed that the interclass correlation (ICC) between instructors was low (ICC=0.14, p=0.52). This means that Mini-CEX test had low intra-rater reliability.  To control the effects of low intra-rater reliability, each instructor was assigned to the determined groups and Mini-CEX results were analyzed and reported separately. There were moderate correlation between pre- and post-test for the two - instructors’ Mini-CEX results in Pearson correlation test (1st instructor, r= 0.56, p=0.05 and 2nd instructor, r=0.62, p=0.04). This revealed moderate interrater reliability for each instructor in this study. 

According to ANOVA test result, there were no significant differences in the mean pre-test scores of the Mini-CEX among the four groups (*F*=0.965, *p=0.417*) while there were significant differences in the mean post-test scores of the Mini-CEX (*F*=4.023, *p=0.012*).

Tukey Post Hoc test revealed a significant difference between the first and second groups, the first and fourth groups, the third and second groups, and the third and fourth groups’ post-test scores of the Mini-CEX, while there was no significant difference between the first and third groups and the second and fourth groups in the post-test scores of the Mini-CEX (Table 1).

**Table 1:** The mean deference of Mini-CEX test among four groups at ANOVA and Tukey HSD post hoc

**Compared** **groups**	***Pre-test***		***Post-test***	
	***Mean*** **±SD** *** ***	***p***	***Mean*** **±SD** *** ***	***p***
**The** ** first group** **/The ** **second group**	0.04±0.90	1.00	5.00±1.69	0.02
**The ** **first group** **/The** ** third group**	0.85±0.88	0.76	0.21±1.66	0.99
**The ** **first group** **/The ** **fourth group**	0.66±0.90	0.88	4.54±1.69	0.04
**The ** **second group** **/The ** **third group**	0.90±0.90	0.74	5.21±1.74	0.02
**The ** **second group** **/The ** **fourth group**	0.61±0.92	0.90	0.46±1.77	0.99
**The ** **third group** **/The ** **fourth group**	1.52±0.90	0.34	4.75±1.69	0.03

The Eta squared among the four groups was 0.19 in ANOVA test, while the Eta squared between the experimental and control groups was 0.17 in the independent t-test. This revealed the small effect size for structured written feedback by cards on the students’ performance at Mini-CEX test based on Cohen classification of the effect size (26).

The mean of the delivered cards is an influential factor in the effect size of the study.  In the experimental groups, 82 cards were delivered to the students. The mean feedback card delivery in the two experimental groups was 2.88±0.65. The instructors completed all cards structured feedback. About 24.39% of the cards had the unstructured written improvement recommendation on their back; however, 75.61% of the cards’ back were left blank. The quantitative content analysis of these recommendations revealed that 75% were classified as “need more practicing” and 25% as “encouraging recommendation” on the cards. These recommendations were not specific though Pearson correlation test results showed that there was no significant relationship between the number of delivered feedback card and students’ performance in post-test (r=0.13, p= 0.52).  

The students’ opinions on the quantity and quality of feedback delivered were analyzed in this study. Most of the students in the four groups were satisfied about the quantity and quality of the feedback they received. In seven items of the self-report questionnaire, there was no statistically significant difference among the four groups in the ANOVA test. According to the ANOVA results, only the two experimental groups perceived the provided feedbacks to be significantly more respectful than the two control groups (*F*=5.27, *p=0.003*). The ANOVA and Post Hoc Tukey test results are shown in Table 2.

**Table 2:** Differences in students opinion about respectfulness of feedback delivery between the four groups according to the contrast analysis (ANOVA) and Tukey HSD post hoc test

**Compared** **groups**	*Mean*±SD* *	*p*
**The** ** first group** **/The ** **second group**	1.08±0.33	0.01
**The ** **first group** **/The** ** third group**	0.20±0.32	0.92
**The ** **first group** ** /The ** **fourth group**	1.01±0.35	0.03
**The ** **second group** **/The ** **third group**	0.88±0.32	0.04
**The ** **second group** **/** **The ** **fourth group**	0.66±0.35	0.99
**The ** **third group** **/The** ** fourth group**	0.89±0.34	0.04

Analysis of the experimental groups’ opinions on the feedback cards indicated that most students agreed or strongly agreed with the size and shape of the cards, the number of cards received from the instructors, and the understandability of the comments on the back of the cards. They are eager to receive feedback cards from their instructors and welcomed their specific comments on the back of the cards. Moreover, 72% of the students suggested the application of this tool for subsequent educational courses. 

## Discussion

The findings presented in this study revealed that the feedback card improved the performance of medical students in outpatient clinic. However, the study had a small effect size; that is, the difference between the groups was statistically important but it was smaller than that of clinical importance.  Similar results are reported in other studies that assessed the effectiveness of feedback card on the students’ performance (27, 28).  Paukert and colleagues used feedback cards to train a number of interns during a 12-week surgery internship. They compared the performance of these students with that of the students of the previous year (as a retrospective control). They reported a greater effect size for feedback card. However, the participants were satisfied with this method and believed that the cards enhanced their skills in history taking, physical examination, and clinical decision-making (12).

The finding of this study revealed that there was a lack of specific recommendation on the back of the cards and most of them were blank. This finding is in accordance with that of Nesbitt and colleagues (9). The instructors’ training on the types of effective feedback is essential in improving the delivery of corrective recommendations (27). Richards and colleagues (14) found out that the number of delivered cards does not matter. Findings here revealed that there was no significant relationship between the number of cards given to each student and the test score at the end of the course. Meaningful content of the feedback for students and specific recommendation note on the card could improve the effectiveness of the feedback cards.  Previous studies showed that instructors gradually provided more specific feedbacks by applying cards (27, 28). It is noteworthy that completing feedback cards might be at first a time-consuming task for the instructors, but it improves the performance over time.

Based on the Kirkpatrick’s model, assessment of the students’ satisfaction is essential in evaluating the effectiveness of educational program (29). The findings presented in this study revealed that the students in the four groups were satisfied with the quality and quantity of the delivered feedback and believed that the feedbacks improved their performance. It must be noted that the verbal feedback with or without structured written feedback had the same effects on the students’ satisfaction.  This may be related to the content of the feedback that was encouraging in all groups. Previous studies revealed that positive feedback content could increase the satisfaction of students on feedback in clinical education (7, 30).

 The two experimental groups believed that the contents of the delivered structured written feedback by cards carry more respect than that of the control groups. Previous studies did not mention this. It may be related to the respect of the students’ confidentiality by delivering  the feedback  content by cards to the students in that busy educational setting. 

## Conclusion

The findings indicated that the structured written feedback improved the performance of medical students more significantly than verbal feedback. The students in the two experimental groups believed that feedback delivery by cards is a more respectful way of feedback provision in outpatient clinic settings. However, further studies must be conducted in other clinical educational courses and on other aspects of clinical performance. 

**Limitations**

The findings indicated that the structured written feedback improved the performance of medical students more significantly than verbal feedback. The students in the two experimental groups believed that feedback delivery by cards is a more respectful way of feedback provision in outpatient clinic settings. However, further studies must be conducted in other clinical educational courses and on other aspects of clinical performance.
